# Recovery of polyphenols from distillery stillage by microwave-assisted, ultrasound-assisted and conventional solid–liquid extraction

**DOI:** 10.1038/s41598-022-07322-0

**Published:** 2022-02-25

**Authors:** Wioleta Mikucka, Magdalena Zielinska, Katarzyna Bulkowska, Izabela Witonska

**Affiliations:** 1 Faculty of Geoengineering, Department of Environmental Biotechnology, University of Warmia and Mazury in Olsztyn, Sloneczna St. 45G, 10-709 Olsztyn, Poland; 2 Faculty of Chemistry, Institute of General and Ecological Chemistry, Lodz University of Technology, Zeromskiego St. 116, 90-924 Lodz, Poland

**Keywords:** Environmental sciences, Environmental chemistry

## Abstract

Recovery of bioactive compounds from distillery waste could be an option for valorizing this waste. This study investigated how the extraction of polyphenols (which have antioxidant activity) from distillery stillage was affected by solvent type and concentration, extraction time, and method of extraction (conventional solid–liquid extraction, CSLE; ultrasound-assisted extraction, UAE; microwave-assisted extraction, MAE). Although recovery was similar with UAE and MAE, 3 min MAE with 80% ethanol and 80% methanol produced the highest yields of total phenolic content (TPC), total flavonoid content (TFC) and phenolic acids. With CSLE, TPC was 2.1–1.8-times lower than with MAE and 1.7–1.4-times lower than with UAE. Increasing the solvent concentration to 100% significantly decreased recovery. Six phenolic acids were recovered (ferulic and p-coumaric acid predominated), which were present mainly in the free form. There was a significant positive correlation between antioxidant activity, as measured with three methods (one based on the hydrogen atom transfer and two based on single electron transfer mechanisms), and phenolic acid content. With MAE and UAE, polyphenols were recovered more efficiently, with 2.1 times and 1.5 times higher antioxidant activity, and with 15 times and 9 times shorter extraction times, respectively, than with CSLE; thus, they can be considered "green" alternatives to CSLE.

## Introduction

The distillery industry makes large contributions to the global economy; however, distilleries generate waste (termed distillery stillage), which contributes significantly to environmental pollution. For every liter of alcohol produced, ca. 8–15 L of by-products are generated which have a low pH and a dark brown color and contain large concentrations of hardly degradable organics and nitrogen^1^. Environmental regulations and increasing understanding of the negative effects of waste discharges have made both treatment and valorization of distillery by-products to produce value-added compounds reasonable options for distillery stillage management.

Distillery stillage contains bioactive products such as polyphenols^2^ with beneficial effects on health due to their antioxidant, antimicrobial and anti-inflammatory properties. Therefore, they can be used in the pharmaceutical, cosmetic, and food industries^3^. On the other hand, at concentrations above 1 g/m^3^, polyphenols may inhibit methanogenesis during anaerobic processing of waste^4^. Therefore, more attention should be paid to the extraction of these bioactive compounds from distillery waste and to limiting their emissions into the natural environment.

Polyphenols differ in terms of chemical structure, which is a very important aspect when choosing solvents and determining the extraction conditions. The most common polyphenols are flavonoids and phenolic acids^5^. The bound forms of phenolic acids account for 80–95% of their total amount and they have linkages with cell wall polymers. To release them from the cell wall matrix, acidic and basic hydrolyses have been performed^3^. In extraction, 80% ethanol was used to recover polyphenols from cereals^6^. As a result, five phenolic acids were obtained with concentrations decreasing in this order: syringic > sinapic > ferulic > p-coumaric > coffee. When extraction was preceded by alkaline hydrolysis, seven acids were obtained with concentrations decreasing in the following order: ferulic > sinapic > vanillic > p-hydroxybenzoic > p-coumaric > syringic > coffee. In another study, the amount of gallic acid obtained after acidic hydrolysis of grape skins without ultrasound irradiation was 4.5 times higher than after extraction without hydrolysis but supported by ultrasounds^7^. With ultrasonic treatment, they recovered the following acids: gallic > p‐coumaric > ellagic > vanillic, whereas after hydrolysis, they obtained acids: gallic > p-hydroxybenzoic > p‐coumaric > syringic > ellagic > vanillic. Hence, the operational conditions of extraction strongly affect the composition of polyphenols.

Conventional solid–liquid extraction (CSLE) uses organic solvents due to their high selectivity for polar compounds^8^. Nevertheless, the search for new extraction techniques has resulted in the development of ultrasound-assisted extraction (UAE) and microwave-assisted extraction (MAE) for polyphenol recovery^9^. However, polyphenols may be degraded at high temperatures^10^ and both the extraction yield and the antioxidant activity of extracts are strongly dependent on the solvent, due to the different antioxidant potentials of compounds with different polarities^11^. Additionally, phenolic compounds can form strong bonds with lignin^2^. Therefore, these extraction techniques should be optimized in terms of types of solvents and operational conditions for each type of waste material.

Antioxidant activity of polyphenols can be monitored by several methods, which are based on the inhibitory action of extracts on free radical activity of compounds used in the antioxidant tests. These tests include the use of 2,2-azinobis (3-ethylbenzothiazoline-6-sulfonic acid) (ABTS), 2,2-diphenyl-1-picrylhydrazyl (DPPH), and iron-reducing antioxidants (FRAP). In general, the total activity of a certain antioxidant can be assessed in terms of hydrogen atom transfer (HAT) or single electron transfer (SET)^12^. In most situations, these two reactions occur simultaneously, and the mechanism is determined by the structure and solubility of the antioxidant, the partition coefficient, and the polarity of the solvent. Because diverse mechanisms are involved in the antioxidant action, to obtain a clear understanding of the relationship between the structure and the activity of the antioxidant, tests based on the HAT and SET are used. ABTS test is based on HAT, whereas DPPH and FRAP tests – on SET mechanisms. Despite the widespread use of these tests, agreement between them depends on the substrate being used. For example, Ou et al.^13^ showed no correlation between FRAP and ABTS results for vegetable samples, while in the case of blueberry fruit these results were highly correlated^14^. Likewise, Awika et al.^12^ showed a strong correlation between ABTS, DPPH, and FRAP results in sorghum and its products. Since the extraction yield and antioxidant activity of polyphenols are strongly dependent on the substrate from which the polyphenols are recovered, such studies are required for each substrate^15^. Because the proportions of phenolic acids vary considerably depending on the type of substrate and the extraction conditions and may affect the antioxidant activity, the solvent should be selected not only for its extraction efficiency, but also regarding the antioxidative properties of the extracted compounds^8^.

Up to now, recovery of polyphenols with simultaneous analysis of their antioxidant activity has been performed with cereals^6^, and regarding wastes, with olive mill wastewaters, vinasses, apple pomace, orange peels, or grape marc^13,15,16^. Based on a literature review, it appears that there is a lack of information on the possibility of recovering polyphenols from distillery stillage. Hence, this study focused on the stillage and investigated the effect of extraction conditions, such as the type and concentration of solvent used, and the time of CSLE, UAE and MAE, on (i) the total polyphenol content (TPC) and total flavonoid content (TFC), (ii) the content and types of the major free and bound phenolic acids in the extracts, and (iii) the antioxidant activity of polyphenols using the ABTS, FRAP and DPPH tests.

## Materials and methods

### Sampling and preparation

Distillery stillage came from a company in north-east Poland that produces concentrated unpurified ethyl alcohol from cereals (wheat and rye). The stillage was characterized by: 43,600–50,400 mg COD/L, 4,345 ± 5 mg N_tot_/L, 280 ± 2 mg P_tot_/L, and 789 ± 3 mg CH_3_COOH/L. After transporting to the lab, the stillage was frozen and then freeze-dried (STERIS Lyovac GT2 freeze dryer with Leybold Trivac vacuum pump).

### Extraction of polyphenols

For freeze-dried stillage samples, three extraction methods were tested: CSLE, UAE and MAE with the use of different solvents and times of extraction. CSLE, UAE and MAE were carried out with aqueous solutions of ethanol (E) and methanol (M) at concentrations of 60–100%. In the series abbreviations, the values after CSLE, UAE and MAE mean the extraction time, while the values after E and M mean the solvent concentration. For each extraction method, the ratio of freeze-dried distillery stillage to solvents was 1:30 (w/v). CSLE was carried out under shaking conditions for 15, 30, 45, 60, and 90 min, with three extraction cycles, at room temperature. For UAE, an ultrasonic bath (InterSonic IS–5,5) with a frequency of 25 kHz was used. The samples were sonicated for 3, 5, 10, 15, and 20 min, at room temperature. MAE was performed using a microwave oven (MARSXpress 240/50, CEM) for 1, 3, 5, 8, and 10 min at 400 W, at 50 °C. After extraction, samples were centrifuged in a 5810R Eppendorf centrifuge for 10 min at 10,000 rpm. In supernatants (extracts), electrical conductivity (EC) (Multi-Range HI8733 conductivity meter), pH (pH meter, HANNA Instruments HI 221), and surface tension (ST) (K100C tensiometer) were measured at room temperature.

### Quantification of TPC and TFC in the extracts

The TPC was measured using the spectrophotometric method with Folin-Ciocalteu (F–C) reagent, according to Singletion et al.^17^ with modifications, and then expressed as Gallic Acid Equivalents (mg GAE/g DM).

The TFC was measured by the aluminum chloride colorimetric method, according to Quettier-Deleu et al.^18^ with modifications, and then expressed as Quercetin Equivalents (mg QUE/g DM).

### Determination of free-soluble and bound-insoluble phenolic acid content by HPLC

To determine the content of free phenolic acids, the supernatant samples were evaporated to dryness in a rotary vacuum evaporator (Vacuum Rotavapor R-210, BUCHI) at below 50 °C. Extraction of free and total phenolic acids was conducted according to the method of Yang et al.^19^ with minor modifications. To determine the free phenolic acids, 20 mL of distilled water (acidified to pH 2) was added to dry extracts to dissolve the precipitate after drying. To determine total phenolic acids, hydrolysis was performed. 1 g of the freeze-dried sample was mixed with 20 mL of 2 M NaOH for 4 h. The samples were acidified to pH 2 with 6 M HCl and centrifuged for 10 min at 10,000 rpm. The supernatants with free and total phenolic acids were extracted four times using 20 mL of diethyl ether. The diethyl ether extracts were dried by a rotary vacuum evaporator and re-dissolved in 1 mL of methanol before being subjected to chromatographic separation. Phenolic acids were determined according to the method described by Chiremba et al.^20^ with modifications. Chromatographic separation was performed by a HPLC (Varian, Australia) fitted with a UV–Vis detector equipped with a Supelcosil C18 column (150 mm × 4.6 mm, 5 μm) with the column flow rate set to 1 mL/min, and the temperature set to 35 °C. The mobile elution phase was acetonitrile/formic acid (99.85/0.15, v/v) (eluent A) and water/formic acid (99.85/0.15, v/v) (eluent B). Phenolic acids were separated for 42 min at a gradient elution flow rate of 1 mL/min with the following multistep gradient: 0–18 min, 1–96% B; 18–35 min, 96–82% B; 35–40 min, 82–75% B. The analysis used wavelengths of 260 nm (p-OH-benzoic, vanillic, syringic acids) at 0–16.50 min and 320 nm (p-coumaric, ferulic, sinapic acids) till the end. All phenolic acid standards were prepared at a concentration of 0.1 mg/mL. Phenolic acids were identified based on the standard curves of the absorption spectra of the reference phenolic acids.

All extraction procedures were first performed with samples of distillery stillage that were spiked with phenolic acid standards. For this procedure, phenolic-acid standard solutions of 0.1 mg/mL were dissolved in acetonitrile, and then 1 mL of these solutions was added to 1 g of freeze-dried stillage. The samples were left for 24 h in the dark.

### Determination of antioxidant capacity

To measure the antioxidant activity of the extracts as their capacity to scavenge free radicals based on the HAT and SET mechanisms, ABTS, DPPH, and FRAP tests were used. The ABTS activity was determined as described by Re et al.^21^ with modifications and expressed as Trolox Equivalents (μmol TE/g DM). The DPPH activity was determined according to Moure et al.^15^ and expressed as Trolox Equivalents (μmol TE/g DM). The FRAP activity was determined according to Benzie and Strain^22^ and expressed as FeSO_4_ equivalents (μmol FeSO_4_/g DM).

### Method validation

The performance of the procedure for identifying phenolic acids in the extracts was assessed using the following indicators: linearity, accuracy, limit of detection (LOD) and limit of quantification (LOQ).

To determine linearity, a standard curve was generated by diluting standard solutions. From the peak-concentration diagram, the coefficients of determination (R^2^) were calculated using linear regression.

To determine the method accuracy, recovery tests were carried out using spiked samples with three concentrations of standard solution (low, medium, high). The recovery efficiency (%) was calculated according to the following equation: R = [(A1 − A2)/A3] × 100, where A1 is the concentration of compounds recovered from the spiked sample, A2 is the concentration of compounds recovered from the sample without spiking, and A3 is the concentration of compounds in the spiked samples.

The LOD and LOQ were calculated based on the analytical curve by selecting appropriate signal-to-noise ratios (3:1 and 10:1, respectively) and using the standard deviation of the response (σ) and the slope of the curve (S). The equations used were as follows: LOD = [(3 × σ)/S] and LOQ = [(10 × σ)/S].

### Data analyses

TFC, TPC, concentrations of phenolic acids, ABTS, FRAP, and DPPH were expressed as mean ± standard deviation. All extractions were performed in duplicate, and analyses were performed in triplicate. Spearman's rank correlation coefficient (r_s_) was used to quantify the relationships between the polyphenol content, antioxidant activities, the physicochemical characteristics of solvents and the extraction type. The correlation matrix was visualized using a correlogram with RStudio Version 1.2.1335 using the “corrplot” package. For statistical analysis, STATISTICA 13.1 (StatSoft) was used, with p ≤ 0.05 defined as significant.

## Results and discussion

### Qualitative assessment of extraction correctness using the standard addition method

The recovery efficiencies of phenolic acids ranged from 52 to 99% (Table 1SM) and decreased in this order: MAE > UAE > CSLE. Using the standard addition method, similar results to those presented in this study were obtained by Robbins and Bean^23^, who reported 69–97% efficiency of polyphenols recovery from wine waste. The efficiency of phenolic acid extraction from non-hydrolyzed and hydrolyzed beer samples was 95.4–104.0% and 94.0–102.9%, respectively^24^. Hence, the present results of the standard addition method confirmed the correctness of the adopted extraction methodology and the procedures, which were performed for quantification of phenolic acids.

### Validation of HPLC method

A wide linear range of standard solutions was used due to the lack of literature reports on the expected phenolic acid concentrations recovered from distillery stillage and the concentration differences between the detected acids (Table 1). The average recoveries were 87.2–96.4% (p-OH benzoic), 95.6–101.7% (vanillic), 79.1–87.7% (syringic), 96.4–103.7% (p-coumaric), 103.7–105.7% (ferulic) and 93.2–102.8% (sinapic). These values were well within acceptable limits (70–110%)^25^, indicating the appropriateness of the methodology; recovery percentages greater than 100% are explained by overlapping sample matrices. The LOD and LOQ ranged from 0.43 to 1.79 µg/L and from 1.42 to 5.99 µg/L, respectively.

**Table 1:** Validation parameters.

Type of acids	Retention time (min)	λ (nm)	Linear range (µg/L)	R^2^	Accuracy level (%)			slope	intercept	LOD (µg/L)	LOQ (µg/L)
					Low	Medium	High				
p-OH benzoic	10.42	260	1–800	0.997	96.4	94.1	87.2	0.0727	0.2475	0.61	2.02
vanillic	12.92	260	1–1000	0.998	101.1	101.7	95.6	0.0459	0.0686	0.95	3.16
syringic	14.42	260	3–800	0.998	84.3	87.7	79.1	0.0205	0.0389	1.79	5.99
p-coumaric	18.29	320	0.7–1000	0.991	103.7	102.4	96.4	0.0804	0.2327	0.49	1.63
ferulic	20.51	320	1–1000	0.999	104.9	103.7	105.7	0.0617	0.0989	0.66	2.19
sinapic	21.08	320	0.5–1000	0.999	95.7	93.2	102.8	0.1032	0.0442	0.43	1.42

### TPC and TFC in distillery stillage extracts

**Figure 1:**
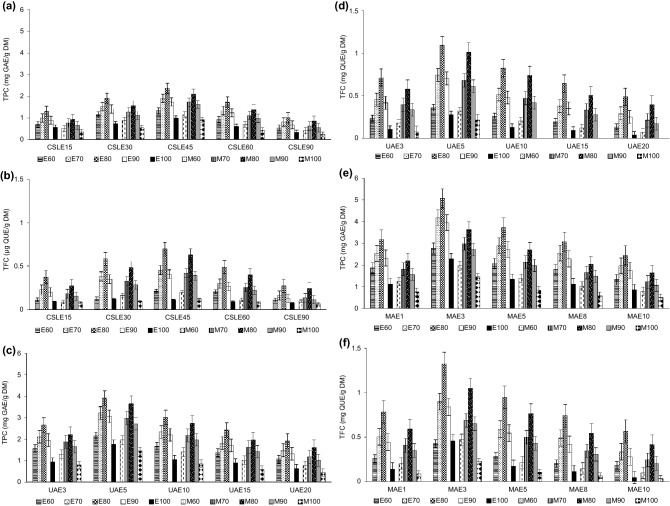
TPC and TFC in distillery stillage extracts obtained with CSLE (**a**,**b**), UAE (**c**,**d**), and MAE (**e**,**f**) for varying extraction time.

TPC and TFC were recovered most efficiently with MAE, followed by UAE, and then CSLE (Fig. 1). TPC was highest when MAE was applied for 3 min with 80% ethanol or 80% methanol (5.07 ± 0.03 mg GAE/g DM or 3.65 ± 0.03 mg GAE/g DM, respectively) (Fig. 1e). With UAE, TPC was slightly lower than with MAE. Recovery with UAE was highest when it was applied for 5 min with 80% ethanol or 80% methanol (3.92 ± 0.02 mg GAE/g DM or 3.65 ± 0.02 mg GAE/g DM, respectively) (Fig. 1c). CSLE was the least efficient method for recovering TPC. The yields were 2.1–1.8-times lower than with MAE and 1.7–1.4-times lower than with UAE. Recovery with CSLE was most effective when it was applied for 45 min with 80% ethanol or 80% methanol (2.36 ± 0.01 mg GAE/g DM or 2.10 ± 0.01 mg GAE/g DM, respectively) (Fig. 1a).

The TFC recovered with MAE during the extraction times decreased in the following order: 3 min > 5 min > 1 min > 8 min > 10 min. With 3 min of MAE, 1.32 ± 0.01 mg QUE/g DM and 1.19 ± 0.02 mg QUE/g DM were obtained with 80% ethanol and 80% methanol, respectively (Fig. 1f). With UAE, the amount of TFC decreased in this order: 5 min > 10 min > 3 min > 15 min > 20 min. With 5 min of UAE, 1.14 ± 0.02 mg QUE/g DM and 1.02 ± 0.01 mg QUE/g DM were obtained with 80% ethanol and 80% methanol, respectively (Fig. 1d). With CSLE, the amount of TFC decreased in this order: 45 min > 30 min > 60 min > 15 min > 90 min. With 45 min of extraction, 0.71 ± 0.02 mg QUE/g DM and 0.63 ± 0.01 mg QUE/g DM were obtained with 80% ethanol and 80% methanol, respectively (Fig. 1b).

In the extraction methods, the shortest times of extraction were selected to be 15 min in CSLE, 3 min in UAE, and 1 min in MAE. Such short contact times are not used commonly to recover polyphenols. However, because the conditions of extraction depend on the sample type^26^, the optimization of extraction time for not recognized so far distillery stillage was started from these times. Unexpectedly, the obtained yields were similar or higher than at the longest time (90 min in CSLE, 20 min in UAE, and 10 min in MAE). To conclude the effect of extraction time on polyphenol yield, TPC and TFC increased significantly when the time was lengthened from 1 to 3 min (MAE), from 3 to 5 min (UAE), or from 15 to 45 min (CSLE). Further extraction lengthening decreased TPC and TFC. Similarly, when compared to CSLE, the increase in extraction yield of TPC was 200% after 5 min and 100% after 15 min of UAE^27^. Although MAE and UAE results in a partial disruption of the polyphenol network into low molecular weight polyphenols, which allows for release the intracellular bioactive compounds in a short time^16,28^, polyphenols may be degraded if they are exposed to microwaves and ultrasounds for too long. This is visible in the present study, in which the TPC and TFC in the extracts decreased when MAE and UAE exceeded 3 min or 5 min, respectively. These decreases may have been caused by the formation of hydrogen peroxide in polyphenol extraction^29^ and the production of free hydroxyl radicals which degrade polyphenols, particularly in the presence of high water content when being exposed to ultrasound^30^. This hypothesis was tested in the present study in a preliminary experiment with known quantities of phenolic acids spiked to the stillage, which was conducted in all extraction variants using the same extraction conditions as in the main experiment. The content of phenolic acids in the extracts decreased when MAE and UAE exceeded 3 min or 5 min, respectively, which indicated degradation of phenolics with the same trend as in the main experiment. In addition, longer extraction times also increased exposure to oxygen and light, which degraded polyphenols^31^. On the other hand, increasing the time of MAE from 5 to 15 min did not change significantly the extracted TPC from spinach waste^32^; however, this relation was valid for a high percentage of ethanol and attributed to the higher solubility of less polar compounds in higher ethanol concentrations.

TPC and TFC were about 1.9–times higher with MAE and UAE than with CSLE because the ultrasounds and microwaves create a faster and stronger mixing effect than that with CSLE. This effect disrupts the cell structures and reduces external resistance, which increases the accessibility of the solvent to the internal particle structure, thus increasing extraction efficiency^33^. Due to temperature and pressure in MAE and UAE, the release of products of the Maillard reaction (during alcohol production) may have been increased. These substances would have reacted with the F–C reagent and aluminum chloride that are used in the TPC and TFC analyses, respectively^34^, resulting in the higher measured TPC and TFC. In the present study, the TPC obtained with MAE and UAE was rather similar, as was the TFC obtained with these two methods. This is because the products of the Maillard reaction can only be present in soluble fractions. During the production of alcohol, cereals are fermented, mixed, and thermally treated, which increases the amount of soluble compounds. Most of these compounds remain in the production environment, while only a small part passes into the stillage. This corresponds to the effect of MAE and UAE on the TPC recovered from vegetable wastes without their previous fermentation. When recovering polyphenols from orange waste, MAE provided ca. fourfold higher extraction yield than UAE because of radiation-inducing rupture of the vegetal cells^32^.

In the present study, the solvent type influenced the polyphenol recovery from distillery stillage (Fig. 1). The use of ethanol recovered more polyphenols than the use of methanol. These results cannot be explained by differences in the presence of oxygen, which may degrade polyphenols, in the extraction solvents. Although the solubility of oxygen in ethanol is higher than in methanol^35^, the solubility difference is not high enough to affect the efficiency of TPC extraction in the present study. It has been found that ethanol recovers flavonoids more efficiently than methanol because it interacts with compounds via non-covalent interactions and promotes their diffusion into solution^36^. On the other hand, Falleh et al.^37^ observed that TPC was higher in the methanolic extract than in the ethanolic extract. These contradictory results can be explained by the fact that different types of polyphenols are extracted by the different solvents due to affinity differences resulting from the different polarities of a target compound and a solvent. Additionally, the TPC and TFC are determined by the substrate from which they are recovered. Various interactions between the components of the substrate and bioactive compounds can change the affinity of these compounds to specific solvents. Hence, determining the effect of a given solvent on the efficiency of polyphenol recovery is still a challenge due to the diversity of binding compounds in the matrix.

In the present study, 80% solutions of ethanol or methanol were most effective in TPC and TFC recovery, which may be due to the solubility of polar carbohydrates and glycosides of secondary metabolites in these solutions. In contrast, TPC and TFC recovery were lowest when 100% ethanol or methanol were used in CSLE, MAE, and UAE (Fig. 1). The fact that pure alcohol solutions were least effective for polyphenol recovery was related to changes in the polarity of the solvent solution. In general, extraction is improved by adding water to solvent, which increases solvent polarity, thus making it more similar to the polarity of phenolics. This similarity intensifies the molecular forces, thus increasing the solubility of the target compounds. This was particularly visible in MAE, in which the extraction yield was improved, as the absorption of microwave radiation is more efficient in polar media^26^. In the present study, the increase in extraction yield with the increasing percentage of ethanol or methanol to 80% and the decrease in the yield when the percentage was higher than 80% indicate that the extracted compounds were of a wide range of polarity. Similarly, when extracting polyphenols from the olive pomace, increasing the ethanol percentage improved the recovery of less polar compounds and decreased the recovery of polar compounds^26^. In addition, because solvents with high polarity can extract compounds with a wide polarity, higher values of TPC in the present study when using solvents of percentages lower than 100% may result from the fact that non-phenolic polar compounds such as carbohydrates and proteins were dissolved during the extraction process, which resulted in increased extraction yields^38^.

The addition of water to alcohol significantly lowered the ST of solvents. With 80% ethanol and methanol, the ST was 21% lower than that of the extracts obtained with pure alcohols. The reduction of the solvent ST increases the mass transfer of phenolic compounds and thus favors the extraction^10^. Additionally, increasing the water content in solvents helps to weaken the hydrogen bonds between the polyphenols and the cell matrix. This allows for better mass transfer of the compounds, and at the same time increases the solubility of the polyphenols, leading to an increase in the extraction yield^14^. However, in the present study, when the alcohol concentration was below 80%, there was a decrease in TPC. An increase in water content in the solvents could have favored the extraction of other compounds from the stillage which do not react with F–C reagent. For example, higher amounts of metal ions (Mn, Cu, Mg, Zn, Na, Ni, Fe, Co) were extracted from wheat seedlings to water extracts than to the ethanol extracts, whereas ethanol extracts had higher antioxidant properties due to polyphenol content than water extracts^39^.

It was noted that the pH affected the TPC and TFC. The extracts obtained with CSLE, UAE, and MAE were acidic (4.16–5.32 pH). In the case of the hydrolysis process, the pH of the reaction mixture was about 2. Acidic conditions hydrolyze cell walls, contributing to the release of phenolics^40^. Our results are consistent with those of other studies, which have reported that lower pH values improve polyphenol extraction^41^. In general, the pH affects the stability and solubility of compounds^40^, which in turn, affects extraction efficiency. More specifically, the OH groups in the phenolic compounds make them particularly susceptible to pH changes. In the present study, acidic conditions could have been particularly important in protection of polyphenols from oxidative degradation in UAE. Acids produce hydrogen ions that stabilize free radicals that may be generated during ultrasonication^42^.

It should be taken into consideration that the F–C reagent is not specific for TPC determination. This reagent can also be reduced by other compounds that may be present abundantly in plant extracts (reducing sugars, ascorbic acid, proteins), distorting the results of TPC^43^. In this sense, measurement of phenols by the F–C method could possibly give too high an estimate of phenolic content. Andres et al.^44^ found that F–C method indicated the phenolic content of 5.42 mg GAE/g in brewer's spent grain. However, HPLC indicated that the phenolic acid content was substantially lower: 178 µg/g. It was indicated that TPC ranges 60–70% of total extractable compounds^27^. Therefore, the polyphenol compounds were separated and quantified by HPLC in the present study.

### Phenolic acid content in distillery stillage extracts

**Figure 2:**
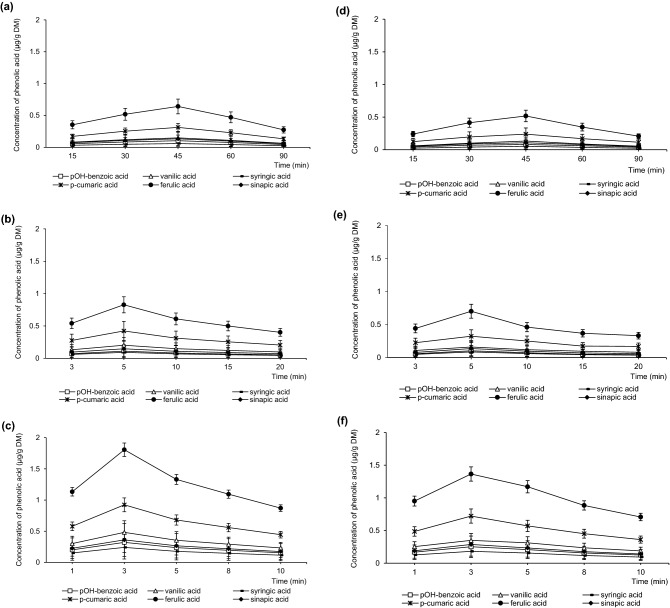
Concentrations of free phenolic acids in extracts obtained with (**a**) CSLE E80, (**b**) UAE E80, (**c**) MAE E80, (**d**) CSLE M80, (**e**) UAE M80, (**f**) MAE M80.

In HPLC, high-quality peak shapes and good separation were obtained, along with minimal baseline variability (Fig. 1SM). In all extracts, six phenolic acids were identified: p-OH benzoic, p-coumaric, vanillic, syringic, sinapic and ferulic. Their concentrations in the most effective extraction variants are shown in Fig. 2; in the other variants—in Fig. 2SM. Ethanol extraction with resulted in a higher content of free phenolic acids than methanol extraction. Both solvents performed more effectively when used in aqueous solutions than in pure form, which was less selective for phenolic acids.

**Figure 3:**
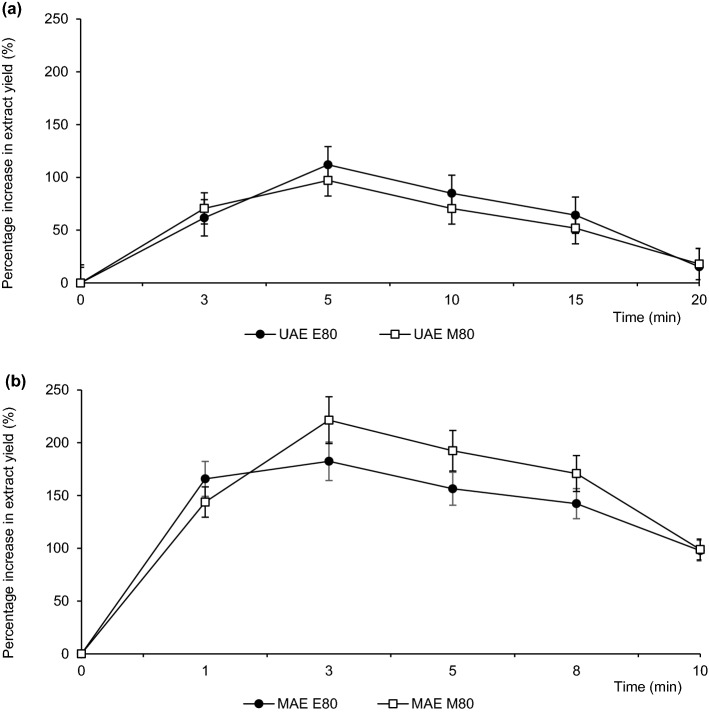
Effect of extraction time on the changes in yields of phenolic acids; (**a**) UAE, (**b**) MAE.

Recovery of free phenolic acids was highest when using 80% ethanol or methanol during MAE for 3 min, with total values of 4.02 ± 0.05 µg/g DM and 3.51 ± 0.04 µg/g DM, respectively (Fig. 2c,f). With UAE, the concentration of phenolic acids was lower, amounting to 2.67 ± 0.04 µg/g DM or 2.49 ± 0.02 µg/g DM for 5-min extraction with 80% ethanol or methanol, respectively (Fig. 2b,e). CSLE was the least effective method for the recovery of phenolic acids (Fig. 2a,d). When using the same solvent at the same concentration, much higher concentrations of phenolic acids were recovered during 3 min of MAE and 5 min of UAE than during 45 min of CSLE. Due to higher extraction selectivity of MAE and UAE^45^, these extraction times gave an increase in extraction yields by 221% (MAE) and 111% (UAE) with ethanol and by 182% (MAE) and 97% (UAE) with methanol, as compared to CSLE (Fig. 3).

So far, no research has been conducted on the phenolic acid recovery from distillery stillage, which comes from cereal processing. Data are available for the overall content of phenolic acids in cereals (e.g. in wheat: 177–257 μg/g DM^46^, 867.38 μg/g DM^34^, or 2612.38 μg/g DM^38^), or in wholemeal flours (4.63 to 7.24 μg/g DM^47^). Treatment and fermentation of the cereals used for alcohol production affect the content of recovered phenolic acids. For example, with spelt and rye breads, sourdough fermentation increased the accumulation of free phenolic acids, while with breads made of common wheat, this type of fermentation reduced their accumulation. Regardless of the fermentation type, free benzoic acids were preferentially accumulated in breads, especially p-OH benzoic and vanillic acids. Ferulic acid is the most abundant acid in maize, rice, wheat, buckwheat, millet, sorghum, rye, and barley^3,20^. Even though Shahidi and Naczk^5^ found that most cereals and cereal products are rich in hydroxycinnamic acids, such as ferulic acid and p-coumaric acid, they reported that concentrations varied, depending on the source from which they were extracted.

In the present study, ferulic and p-coumaric acids predominated, accounting for 42% to 48% and 23% to 25% of all phenolic acids detected, respectively, depending on the extraction variant employed (Fig. 2). Next, vanillic and sinapic acids accounted for approx. 10–12% and 5–8% of this fraction, respectively. The sum of benzoic acids (p-OH benzoic and syringic) in the stillage was approx. 5–7%. This is similar to what was reported in other studies involving wheat, rye and sorghum, as well as flour and bread produced from these cereals, in which the content of ferulic, p-coumaric and vanillic acids was highest^48^. The phenolic acids that were detected in the stillage extracts in the present study are considered having some properties that are useful for the food, pharmaceutical and cosmetic industries. Due to low toxicity, ferulic acid is widely used as a cross-linking agent in the preparation of food gels and edible films, as a skin protection agent and as an ingredient in sports nutrition. P-coumaric acid has a bactericidal effect^49^. Vanillic acid is used as a fragrance in the food and cosmetics industry^50^. Sinapic acid acts as a powerful oxidant scavenger, thus protecting cellular components^51^.

Although many phenolic acids in cereal products are bound to cell wall materials, particularly polysaccharides, lignins and suberin, in the present study, only a small fraction of phenolic acids existed in a bound state. The concentration of free phenolic acids reached 4.02 ± 0.05 µg/g DM, and these acids constituted up to 92% of the total phenolic acids. The average concentration of total phenolic acids was 4.36 ± 0.60 µg/g DM. High contribution of free phenolic acids may be caused by the activity of phenolic acid esterases in fermentation and processing of cereals during the alcohol production process, when phenolic acids bound to the cell wall are released^47^. Additionally, during cereal processing, most of the phenolic acids pass into the product, while only a small part remains in the by-products. Moreover, heat treatment can cause changes in the phenolic content. In the present study, short duration of the supporting factor (UAE shorter than 5 min and MAE shorter than 3 min) resulted in the recovery of only a part of bound phenolic acids. However, with longer durations (UAE longer than 5 min and MAE longer than 3 min), the content of phenolic acids decreased in proportion with the increase in time (Fig. 2). These differences were most pronounced in the cases of ferulic acid and p-coumaric acid recovery: the respective amounts that were recovered were 2.23 and 1.94 times higher with 3-min MAE than with 10-min MAE, and 1.98 and 1.89 times higher with 5-min UAE than with 20-min UAE. Although it was reported that MAE or UAE improves the recovery, literature reports on the effect of time of exposition to this treatment are inconsistent. This is because these changes in phenolic acid content may result from the oxidative degradation of these acids, including enzymatic browning, the release of free acids from conjugated forms or the formation of phenolic substances with complex structures from related compounds, such as proteins, tannins and anthocyanins^52^.

### Antioxidant activity of polyphenols in distillery stillage extracts

The antioxidant activity of recovered phenolic compounds depends largely on the type, concentration, and polarity of the solvent used to recover them. Due to different mechanisms of reaction and conditions of these test, limitations of each test, and their ability to determine various types of antioxidants, the DPPH, ABTS and FRAP tests were used in the experiment. The antioxidant activities of the extracts obtained with MAE for 3 min with 80% ethanol were the highest, as indicated by the ABTS (26.73 ± 1.81 μmol TE/g DM), DPPH (5.57 ± 0.38 μmol TE/g DM) and FRAP (3.71 ± 0.26 μmol FeSO_4_/g DM). When MAE was used for 3 min with 80% methanol, the extract had antioxidant activities that were somewhat lower (Table 2 and 2SM). The antioxidant activity was lowest with CSLE for 90 min with 100% ethanol and methanol. Higher antioxidant activity of ethanolic extracts than that of methanolic extracts corresponded with the higher TPC in ethanolic extracts. This relation between TPC and antioxidant activity may result from the absence of any potential interfering (absorbing) species which do not have antioxidant activity^53^. However, this relation is dependent on type of substrate and target compounds. Falleh et al.^37^ reported that although TPC was higher in the methanolic extract than in the ethanolic extract, the ethanolic extract had higher antioxidant activity.

**Table 2:** Antioxidant activities of distillery stillage extracts obtained with 80% solvents.

Type of extraction	Antioxidant assays			Type of extraction	Antioxidant assays			Type of extraction	Antioxidant assays		
	ABTS (μmol TE/g DM)	DPPH (μmol TE/g DM)	FRAP (μmol FeSO_4_/g DM)		ABTS (μmol TE/g DM)	DPPH (μmol TE/g DM)	FRAP (μmol FeSO_4_/g DM)		ABTS (μmol TE/g DM)	DPPH (μmol TE/g DM)	FRAP (μmol FeSO_4_/g DM)
CSLE15 E80	8.46 ± 1.88	2.41 ± 0.37	1.56 ± 0.10	UAE3 E80	14.60 ± 1.22	4.16 ± 0.26	2.70 ± 0.22	MAE1 E80	24.26 ± 1.56	5.05 ± 0.28	3.37 ± 0.18
CSLE15 M80	8.19 ± 1.57	2.34 ± 0.31	1.50 ± 0.09	UAE3 M80	14.12 ± 1.34	3.67 ± 0.38	2.45 ± 0.17	MAE1 M80	17.64 ± 0.89	4.04 ± 0.22	2.53 ± 0.23
CSLE30 E80	9.44 ± 1.92	2.72 ± 0.35	1.85 ± 0.18	UAE5 E80	16.67 ± 1.10	4.54 ± 0.41	2.96 ± 0.28	MAE3 E80	26.73 ± 1.81	5.57 ± 0.38	3.71 ± 0.26
CSLE30 M80	9.07 ± 1.67	2.59 ± 0.24	1.71 ± 0.21	UAE5 M80	15.48 ± 1.09	4.34 ± 0.31	2.75 ± 0.21	MAE3 M80	25.33 ± 0.78	5.27 ± 0.31	3.52 ± 0.25
CSLE45 E80	10.84 ± 2.01	2.95 ± 0.21	1.93 ± 0.19	UAE10 E80	14.99 ± 1.42	4.32 ± 0.28	2.94 ± 0.26	MAE5 E80	27.54 ± 0.65	5.74 ± 0.29	3.83 ± 0.29
CSLE45 M80	10.07 ± 1.79	2.82 ± 0.29	1.79 ± 0.19	UAE10 M80	14.39 ± 1.27	4.11 ± 0.21	2.71 ± 0.17	MAE5 M80	23.22 ± 0.84	4.83 ± 0.17	3.22 ± 0.15
CSLE60 E80	8.25 ± 1.56	2.34 ± 0.26	1.58 ± 0.15	UAE15 E80	14.23 ± 1.58	4.03 ± 0.27	2.72 ± 0.16	MAE8 E80	23.52 ± 0.74	4.90 ± 0.24	3.27 ± 0.11
CSLE60 M80	7.97 ± 1.42	2.23 ± 0.18	1.49 ± 0.14	UAE15 M80	13.74 ± 1.44	3.84 ± 0.34	2.57 ± 0.11	MAE8 M80	22.24 ± 0.63	4.63 ± 0.29	3.09 ± 0.04
CSLE90 E80	7.01 ± 1.68	1.89 ± 0.35	1.41 ± 0.11	UAE20 E80	12.74 ± 0.96	3.44 ± 0.18	2.56 ± 0.07	MAE10 E80	21.22 ± 0.87	4.42 ± 0.27	2.95 ± 0.08
CSLE90 M80	6.52 ± 1.86	1.77 ± 0.38	1.28 ± 0.08	UAE20 M80	11.86 ± 1.03	3.22 ± 0.14	2.32 ± 0.02	MAE10 M80	19.17 ± 0.57	3.99 ± 0.38	2.66 ± 0.11

In all extracts, the ABTS assay indicated significantly higher antioxidant activity than the DPPH assay, which was followed by the FRAP assay. The predominance of antioxidant activity shown by the ABTS and DPPH over that shown by the FRAP may be due to the predominance of phenolic compounds that are efficient at scavenging radicals and smaller quantity of non-phenolic compounds that may be able to reduce FRAP, account for the reducing power but are not so efficient at scavenging radicals^53^. Similarly, Yu et al.^46^ reported that the ABTS assay indicated that the activity of wheat bread extract was 10–20-times higher than what the DPPH assay indicated. Smuda et al.^54^ found that the ABTS test indicated higher activity in by-products from wheat, rice and corn than the FRAP test. One observation for this is the fact that each test has a different mechanism, based on HAT (ABTS) or SET (DPPH and FRAP). The extract characteristics, including EC and pH, substantially influences these mechanisms of antioxidant activity^55^. In the present study, the EC of the extracts decreased as the solvent concentration was increased. For example, the EC in the extracts obtained from MAE3 was 7.5, 7.4, 7.2, 7.1 and 7.0 S/m for 60, 70, 80, 90 and 100% ethanol, respectively. The ABTS, DPPH and FRAP test values were correlated with EC; however, irrespective of these EC changes, values of ABTS test were higher than values of DPPH and FRAP tests in each extract. Therefore, EC changes in the stillage extracts did not explain these differences.

A second and related explanation for the differences in antioxidant activity measured with the three assays is the acidic extraction environment. Electron transfer reactions depends on the ionization potential of the active functional group and thus depends on the pH. The acidic environment inhibits the deprotonation of functional groups of polyphenolic compounds, which results in a reduced ability to donate electrons because deprotonation generally only lowers the ionization potential (SET) and not the bond dissociation enthalpy (HAT). Hence, it can be concluded that electron transfer is not the dominant mechanism of the antioxidant activity of the bioactive compounds examined in this study. However, it is worth noting that the mechanism of antioxidant action may change as the pH value increases. Thus, in this study, the environment was not conducive to the deprotonation of polyphenolic compounds, so H• donation became easier than electron donation. Therefore, the values of antioxidative activity obtained with the DPPH and FRAP tests were significantly lower than the values obtained with the ABTS test.

The reason for significantly different values of the antioxidant activity tests results from their principles. From three tests examined in this study, the values of the ABTS test were the highest because this test is used to measure the antioxidant capacity of both hydrophobic and hydrophilic samples, ABTS reagent is soluble in both aqueous and organic solvents, and the reaction of ABTS with flavonoids produces products with stronger antioxidant properties and faster reacting with the radical than other phenolic compounds^32^. The DPPH test does not allow the determination of hydrophilic antioxidants. Regarding the FRAP test, some compounds with antioxidant properties do not show reducing capacity towards FRAP reagent, and the formed iron ions can participate in the radical production in the presence of H_2_O_2_, which also distorts the result. That is why the values of the FRAP test were the lowest in this study.

### Correlations between TPC, TFC, phenolic acids, antioxidant activity and extraction conditions

**Figure 4:**
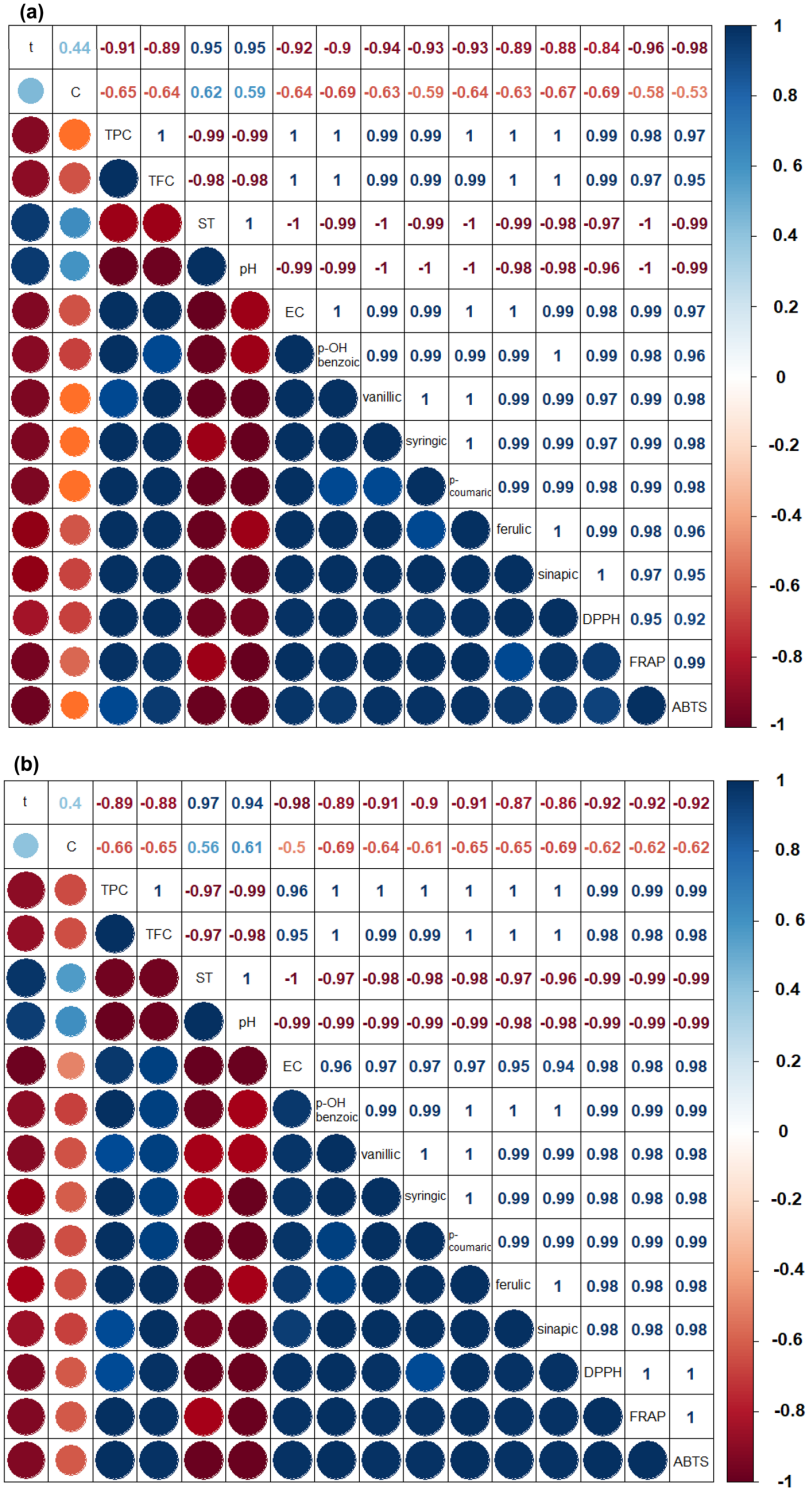
Correlations between TPC, TFC, phenolic acid content, antioxidant activities and extraction parameters; (**a**) UAE with ethanol, (**b**) MAE with ethanol. Positive correlations are displayed in blue and negative correlations in red. Color intensity and the size of the circles are proportional to the correlation coefficients. Values of Spearman’s rank correlation coefficient above 0.6 are described as a strong correlation; t—extraction time, C—solvent concentration.

Antioxidant activity (ABTS) was strongly correlated with TPC and TFC (r_s_ ≥ 0.95), indicating that TPC and TFC were mainly responsible for the activity of the extracts (Fig. 4). Antioxidant activity (ABTS) was positively correlated with the contents of p-OH benzoic (0.96), vanillic (0.97), syringic (0.96), ferulic (0.95), p-coumaric (0.98) and sinapic acid (0.98). The antioxidant effect of these extracts is attributed to the strong free radical scavenging effect of their free hydroxyl groups. This study showed that phenolic acid content was significantly negatively correlated with the extraction time, pH and ST. Their content was also negatively correlated with the concentration of the solvent, although these correlations were not statistically significant.

The relationship between antioxidant activity and polyphenol content can be explained in many ways. The synergy between the antioxidants makes the activity depend not only on the phenolic acid content but also on the structure and interactions between them. This is the reason why extracts with similar concentrations of TPC, TFC, and phenolic acids may differ in their antioxidant activity. Numerous studies of the antioxidant properties of phenolic acids suggested that they are related to the chemical structure of acids, more precisely to the number of hydroxyl groups in the molecule and the degree of their esterification. Balasundram et al.^56^ noted that the antioxidant activity of phenolic acids increases with the number of hydroxyl groups. In compounds with a single hydroxyl group, the antioxidant activity is further enhanced by the presence of one or two methoxy groups on the ring, which is why sinapic acid with two methoxy groups is more active than ferulic acid with one methoxy group, which in turn is more active than p-coumaric acid (containing one hydroxyl group)^57^. The absence of methoxy groups creates a destabilized radical that is not sufficiently stable for p-coumaric acid to be an effective scavenger. In the present study, it was observed that ferulic and p-coumaric acids dominated among the acids in each extract. Along with an increase in their concentration, the antioxidant activity also increased. Therefore, the highest antioxidant activities were observed in ethanol extracts from 3-min MAE. Thus, the results clearly indicate that ferulic and p-coumaric acids have a strong antioxidant effect. It has been shown that the presence of a single CH bond, a double CH bond and a single COOH bond in hydroxycinnamic acids (ferulic, p-coumaric and sinapic) provides a greater ability to donate H and subsequently stabilize radicals than the carboxylate group in hydroxybenzoic acids (p-OH benzoic, vanillic, syringic). Therefore, the type of phenolic acids and hence their chemical/molecular structures exhibit different antioxidant properties. Additionally, the presence of a carboxyl group in ferulic acid facilitates the anchoring of ferulic acid in the lipid bilayer, providing effective protection against lipid peroxidation^58^. Therefore, special attention should be paid to the recovery of phenolic acids from distillery stillage due to its high concentration of ferulic acid, which has a high antioxidant activity.

### Economic aspects

Although distillery stillage extracts contain lower concentrations of phenolic compounds per gram of dry mass than raw plant materials, this waste is produced in billions of tons per year resulting in the extracted phenolic compounds up to tens of thousands of kilograms. Taking into account the economic aspects of using solvents to valorize the stillage, the production costs of solvents that were used in the present study are quite low because the boiling points of ethanol (78.4 °C) and methanol (64.7 °C) allow them to evaporate quickly, which shortens the processing time. Additionally, ethanol, which gave higher extraction yields than methanol in the present study, has been recognized as GRAS (Generally Recognized As Safe) solvent, hence its extracts can find applications in the production of functional foods, dietary supplements, animal feed, or cosmetics^59^. In the present study, the use of 80% solution of ethanol allowed the highest recovering of compounds with antioxidant activity, which, when compared with the use of pure ethanol, increased the economic viability of the obtained extracts. In preliminary studies (data not shown), the solid-to-solvent ratio was selected to be 1:30 (w/v), which is an optimal value considering not only extraction yield but also the cost of purification and concentration of extracts. When comparing the extraction methods, polyphenol recovery yield was about 1.3 times higher with 3-min MAE than with 5-min UAE. These extractions consumed 20.0 Wh (MAE) and 45.2 Wh (UAE) of energy. However, other studies reported that despite higher extraction yields in MAE than in UAE, UAE was suggested as a technique for a future scaling-up evaluation due to its simplicity and investment and operational costs^32^. Therefore, implementing these extraction techniques on an industrial scale will need the assessment of other factors determining the economic and environmental impact. To further increase the profitability of the examined method of distillery stillage valorization, anaerobic treatment of post-extracted stillage for biogas production should be considered.

## Conclusions

The study suggests that distillery stillage can be considered a rich source of natural antioxidants for further use. Optimizing extraction procedure allowed high-value extracts to be obtained with the use of solvents available in any distillery. Phenolic acids were present mainly in the free form (92% of the total content), which allows to avoid pre-treatment or adding harmful reagents for hydrolysis before their recovery. The TFC, TPC, and phenolic acids content depended on the extraction conditions and the solvent type and concentration. With each extraction method, 80% ethanol yielded extracts that were the richest in polyphenols (up to 5.07 ± 0.03 mg GAE/g DM), which contributed to the observed antioxidant activity (26.73 ± 1.81 µmol TE/g DM). Due to providing 2.1 and 1.7 times more efficient recovery and 2.1 and 1.5 times higher antioxidant activity at lower solvent concentrations than CSLE, MAE and UAE can be considered “green” alternatives to CSLE and recommended for the recovery of polyphenols from distillery stillage.

Future studies on recovering polyphenols from distillery stillage should focus on the improvement of recovery efficiency (in terms of profitability, time, and energy consumption) to shorten the reaction time and decrease the amount of solvent used. The development of cheap and green extraction procedures, including searching for waste-derived solvents, that will allow for obtaining a valuable extract rich in bioactive compounds is still a challenging issue. In addition, further studies should focus on the methods of purification and concentration of recovered phenolic compounds to obtain products that will be applicable in the pharmaceutical, cosmetic, and food industries. To confirm the favorable applicability of these products for commercial applications, in vivo studies are needed.

## Supplementary Information

Supplementary Information.
