# Supplementary material for: Recovery of polyphenols from distillery stillage by microwave-assisted, ultrasound-assisted and conventional solid–liquid extraction
Source: Sci Rep. 2022 Feb 25;12:3232. doi: 10.1038/s41598-022-07322-0 (PMC8881464; doi:10.1038/s41598-022-07322-0)
Supplement: Supplementary file 1 — Supplementary Information. [file 41598_2022_7322_MOESM1_ESM.docx]

**Recovery of polyphenols from distillery stillage by microwave-assisted, ultrasound-assisted and conventional solid-liquid extraction**

Wioleta Mikucka^a*^, Magdalena Zielinska^a^, Katarzyna Bulkowska^a^, Izabela Witonska^b^

^a^University of Warmia and Mazury in Olsztyn, Faculty of Geoengineering, Department of Environmental Biotechnology, Sloneczna St. 45G, 10-709 Olsztyn, Poland

^b^Lodz University of Technology, Faculty of Chemistry, Institute of General and Ecological Chemistry, Zeromskiego St. 116, 90-924 Lodz, Poland

*Corresponding author

*E-mail address:* wioleta.mikucka@uwm.edu.pl (W. Mikucka).

**Supplementary Material**


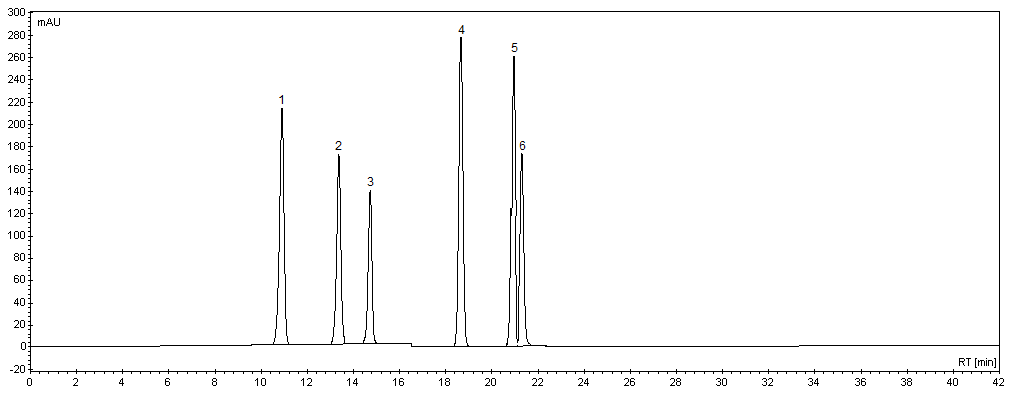


**Figure 1SM.** HPLC chromatogram of phenolic acid standards at 260 nm, peaks: 1 – p-OH benzoic acid, 2 – vanillic acid, 3 – syringic acid, and at 320 nm, peaks: 4 – p-coumaric acid, 5 – ferulic acid, 6 – sinapic acid


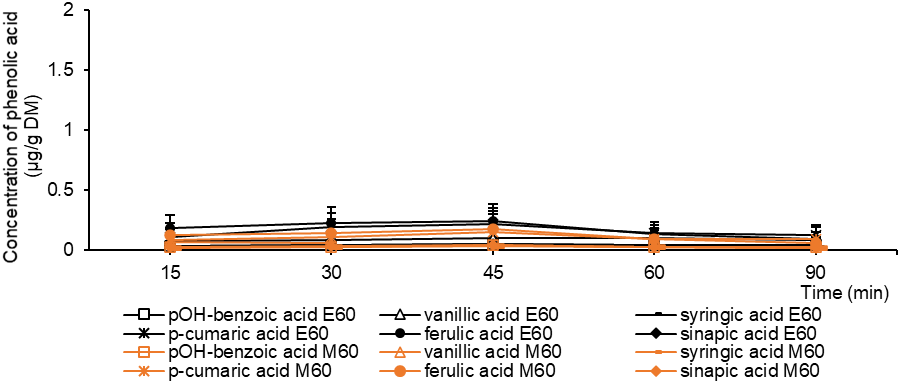


**(a)**


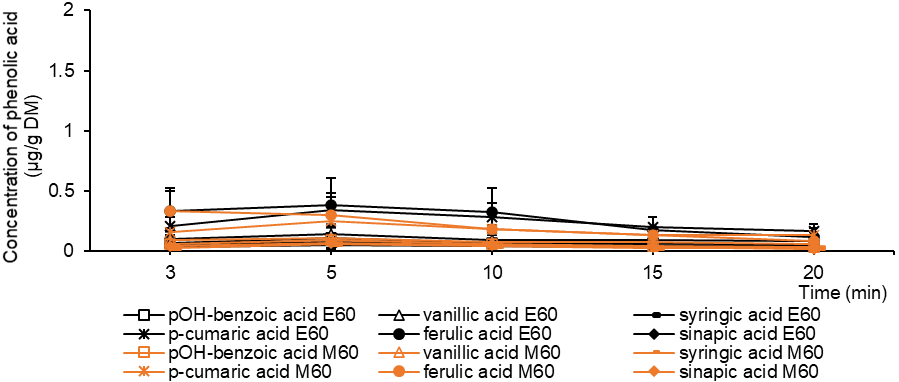


**(b)**


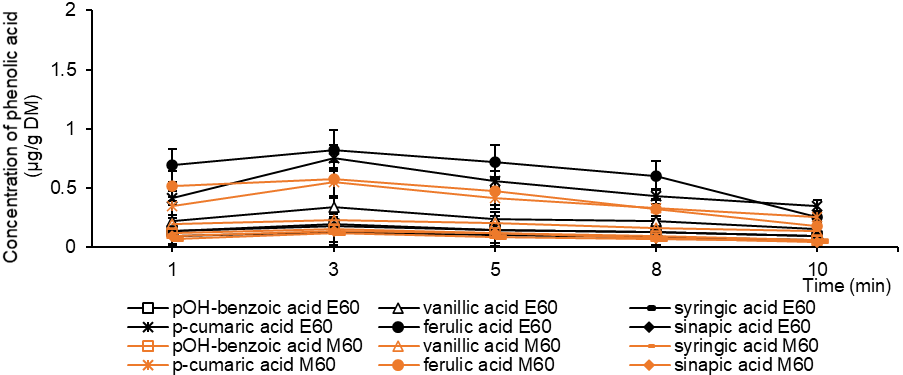


**(c)**


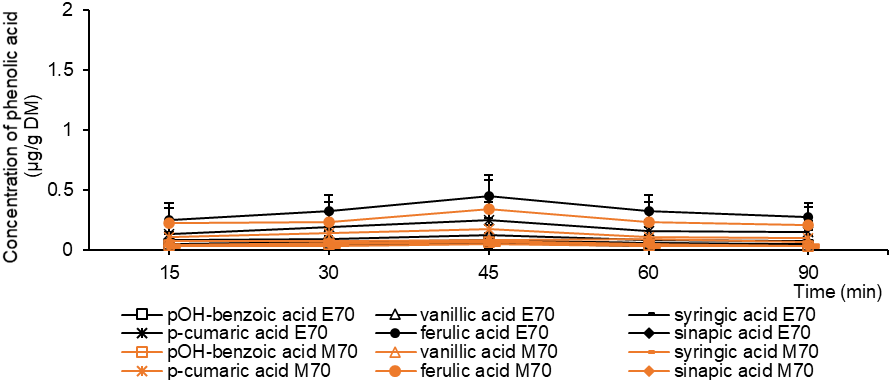


**(d)**


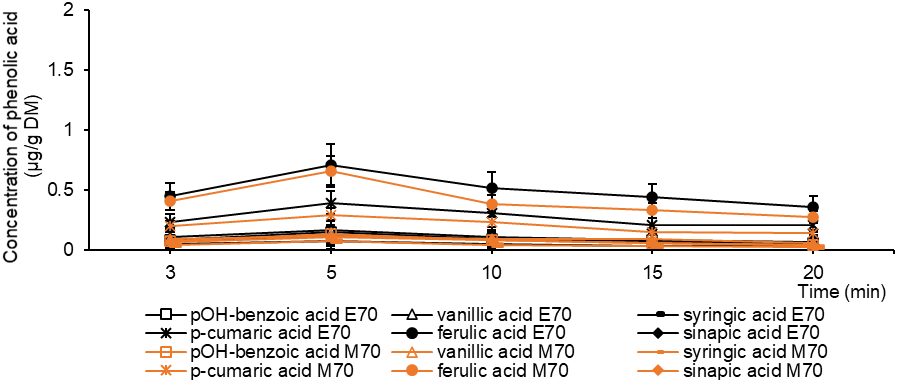


**(e)**


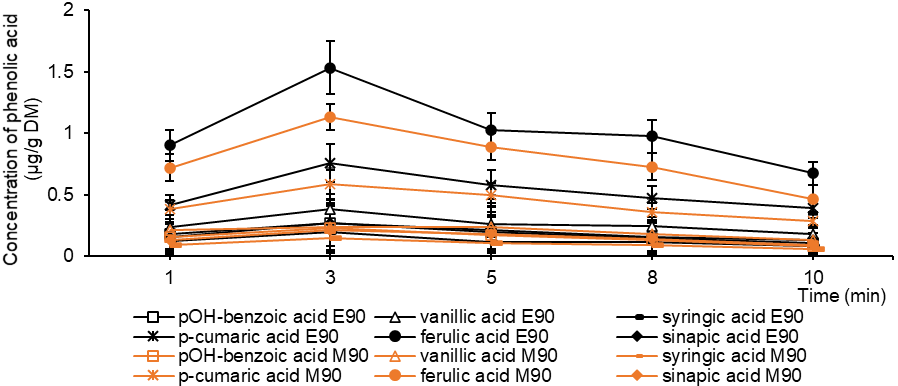


**(i)**


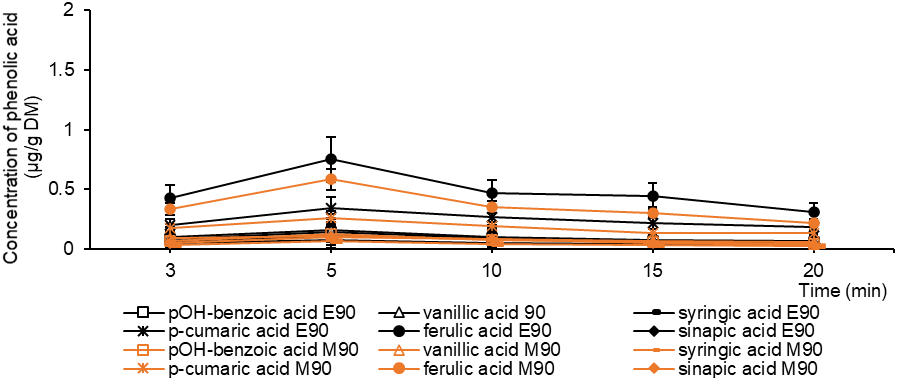


**(h)**


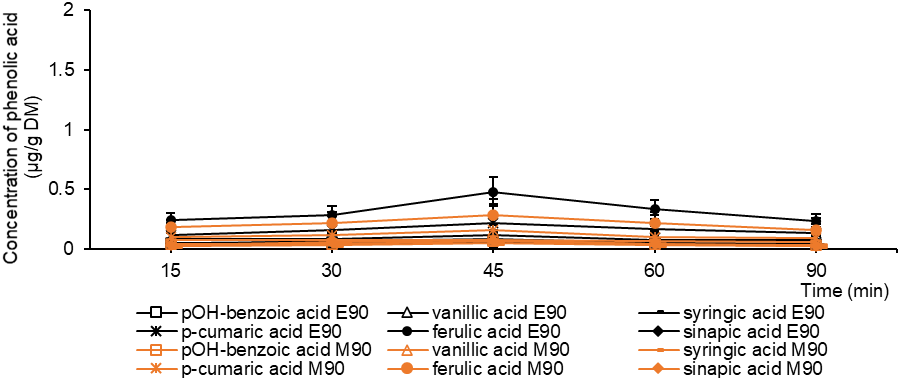


**(g)**


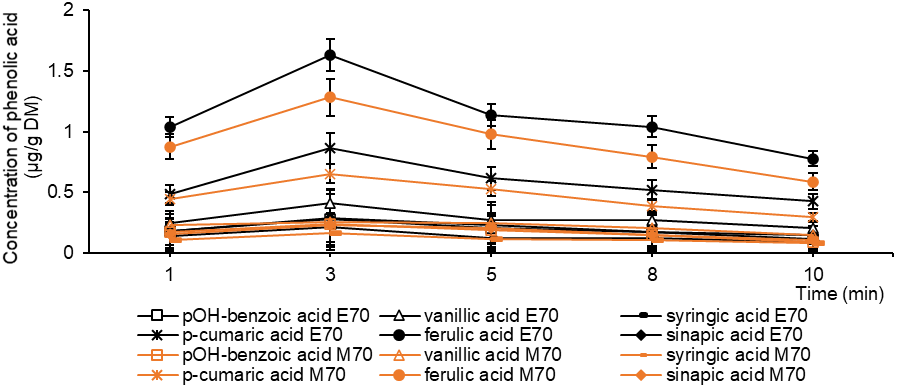


**(f)**


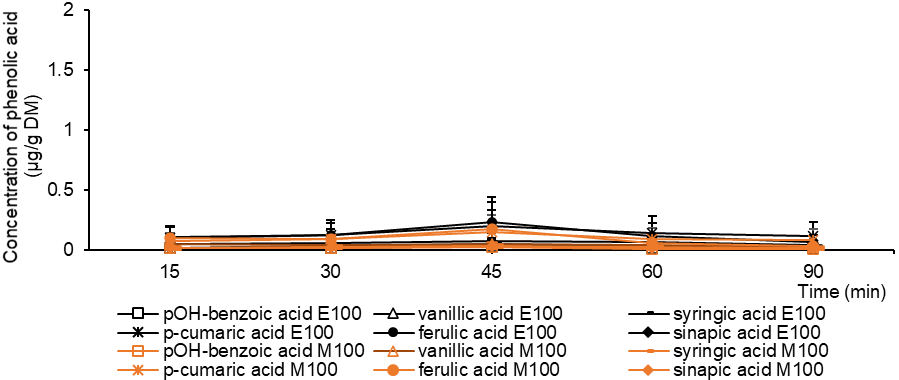


**(j)**


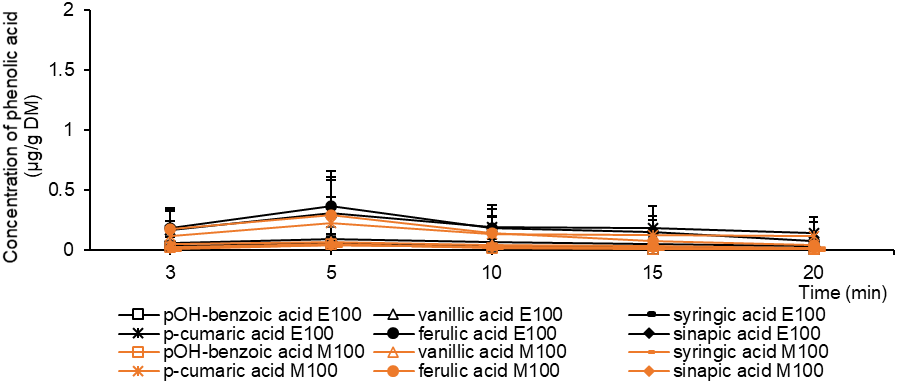


**(k)**


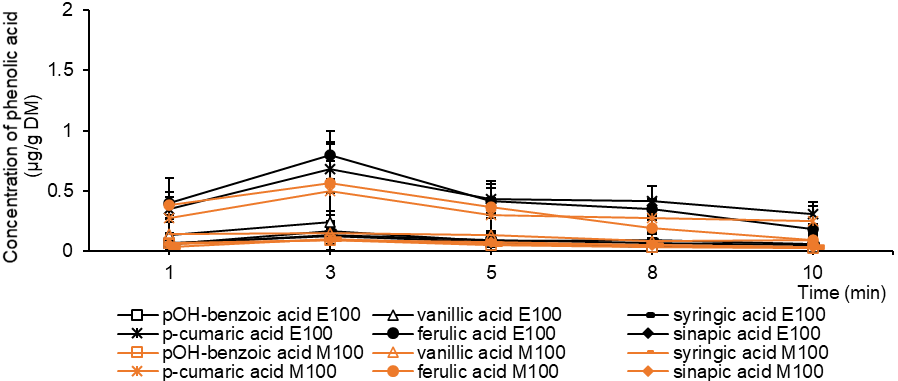


**(l)**

**Figure 2SM.** Concentrations of free phenolic acids in extracts obtained with **(a)** CSLE E60 and M60, **(b)** UAE E60 and M60, **(c)** MAE E60 and M60, **(d)** CSLE E70 and M70, **(e)** UAE E70 and M70, **(f)** MAE E70 and M70, **(g)** CSLE E90 and M90, **(h)** UAE E90 and M90, **(i)** MAE E90 and M90, **(j)** CSLE E100 and M100, **(k)** UAE E100 and M100, **(l)** MAE E100 and M100

**Table 1SM.** Efficiency of recovery of phenolic acids from distillery stillage using the standard addition method

| Concentration of alcohol (%) | Type of extraction | | | | | | | | | | | | | | |
| --- | --- | --- | --- | --- | --- | --- | --- | --- | --- | --- | --- | --- | --- | --- | --- |
|  | CSLE15 | CSLE30 | CSLE45 | CSLE60 | CSLE90 | UAE3 | UAE5 | UAE10 | UAE15 | UAE20 | MAE1 | MAE3 | MAE5 | MAE8 | MAE10 |
| E60 | 68.86 | 69.85 | 67.93 | 58.77 | 54.09 | 76.53 | 79.31 | 69.20 | 62.80 | 57.37 | 76.07 | 81.23 | 73.93 | 66.70 | 60.92 |
| E70 | 73.99 | 77.03 | 70.56 | 61.91 | 54.96 | 80.98 | 85.58 | 75.69 | 68.52 | 61.73 | 84.01 | 89.03 | 78.76 | 70.65 | 63.92 |
| E80 | 77.80 | 81.88 | 73.42 | 63.90 | 55.60 | 89.39 | 96.27 | 81.30 | 71.38 | 64.45 | 91.85 | 99.14 | 84.63 | 73.99 | 66.93 |
| E90 | 70.74 | 73.92 | 69.67 | 60.23 | 54.50 | 78.92 | 82.80 | 71.89 | 65.36 | 59.60 | 81.00 | 85.91 | 75.23 | 68.51 | 62.80 |
| E100 | 66.77 | 67.98 | 65.68 | 55.04 | 53.93 | 73.03 | 76.45 | 67.49 | 61.85 | 56.57 | 70.68 | 76.33 | 71.26 | 65.68 | 59.03 |
| M60 | 59.49 | 67.52 | 57.69 | 54.19 | 52.85 | 71.83 | 75.28 | 64.52 | 61.05 | 55.35 | 72.63 | 77.46 | 72.38 | 66.02 | 59.91 |
| M70 | 66.70 | 70.44 | 62.10 | 59.34 | 53.91 | 77.11 | 81.06 | 68.68 | 66.57 | 59.49 | 79.72 | 85.86 | 78.68 | 69.43 | 62.41 |
| M80 | 69.77 | 72.59 | 66.21 | 60.64 | 54.56 | 81.86 | 87.22 | 73.52 | 69.91 | 61.04 | 84.65 | 90.28 | 81.35 | 72.79 | 64.44 |
| M90 | 64.15 | 69.90 | 60.41 | 57.16 | 53.61 | 75.74 | 78.35 | 65.77 | 64.24 | 57.19 | 76.65 | 81.23 | 75.52 | 67.55 | 61.36 |
| M100 | 57.06 | 56.72 | 55.34 | 52.45 | 52.05 | 67.48 | 71.57 | 62.54 | 59.69 | 54.79 | 68.48 | 73.68 | 65.87 | 65.16 | 58.53 |

**Table 2SM.** Antioxidant activities of distillery stillage extracts obtained with 60%, 70%, 90%, and 100% solvents

| Type of extraction | Antioxidant Assays | | | Type of extraction | Antioxidant Assays | | | Type of extraction | Antioxidant Assays | | |
| --- | --- | --- | --- | --- | --- | --- | --- | --- | --- | --- | --- |
|  | ABTS (μmol TE/g DM) | DPPH (μmol TE/g DM) | FRAP (μmol FeSO_4_/g DM) |  | ABTS (μmol TE/g DM) | DPPH (μmol TE/g DM) | FRAP (μmol FeSO_4_/g DM) |  | ABTS (μmol TE/g DM) | DPPH (μmol TE/g DM) | FRAP (μmol FeSO_4_/g DM) |
| CSLE15 E60 | 8.18±1.23 | 2.27±0.52 | 1.38±0.24 | UAE3 E60 | 14.11±1.44 | 3.91±0.63 | 2.38±0.44 | MAE1 E60 | 21.38±1.88 | 4.46±0.88 | 2.97±0.51 |
| CSLE15 E70 | 8.38±1.12 | 2.40±0.48 | 1.52±0.27 | UAE3 E70 | 14.45±1.46 | 4.13±0.69 | 2.61±0.46 | MAE1 E70 | 23.51±1.92 | 4.90±0.96 | 3.27±0.68 |
| CSLE15 E90 | 8.33±1.14 | 2.36±0.46 | 1.49±0.25 | UAE3 E90 | 14.36±1.45 | 4.07±0.71 | 2.57±0.45 | MAE1 E90 | 23.17±1.86 | 4.83±0.91 | 3.22±0.66 |
| CSLE15 E100 | 8.09±1.19 | 2.18±0.39 | 1.29±0.21 | UAE3 E100 | 13.95±1.41 | 3.76±0.60 | 2.23±0.41 | MAE1 E100 | 20.10±1.41 | 4.19±0.74 | 2.79±0.41 |
| CSLE15 M60 | 7.93±1.08 | 2.13±0.35 | 1.36±0.24 | UAE3 M60 | 13.67±1.38 | 3.34±0.54 | 2.23±0.38 | MAE1 M60 | 16.04±1.32 | 3.67±0.63 | 2.34±0.31 |
| CSLE15 M70 | 8.12±1.15 | 2.27±0.38 | 1.47±0.27 | UAE3 M70 | 13.99±1.42 | 3.56±0.56 | 2.38±0.43 | MAE1 M70 | 17.11±1.48 | 3.92±0.62 | 2.58±0.38 |
| CSLE15 M90 | 8.06±1.12 | 2.22±0.34 | 1.44±0.22 | UAE3 M90 | 13.90±1.43 | 3.45±0.51 | 2.30±0.42 | MAE1 M90 | 16.57±1.38 | 3.82±0.55 | 2.49±0.35 |
| CSLE15 M100 | 7.85±1.14 | 2.06±0.31 | 1.28±0.25 | UAE3 M100 | 13.55±1.36 | 3.23±0.48 | 2.16±0.36 | MAE1 M100 | 15.50±1.33 | 3.55±0.59 | 2.21±0.29 |
| CSLE30 E60 | 9.05±1.22 | 2.55±0.39 | 1.55±0.29 | UAE5 E60 | 16.24±1.52 | 4.25±0.71 | 2.54±0.44 | MAE3 E60 | 25.12±1.96 | 5.24±0.94 | 3.49±0.34 |
| CSLE30 E70 | 9.30±1.35 | 2.70±0.42 | 1.82±0.31 | UAE5 E70 | 16.49±1.58 | 4.50±0.76 | 2.89±0.48 | MAE3 E70 | 26.20±2.03 | 5.46±0.97 | 3.64±0.39 |
| CSLE30 E90 | 9.25±1.23 | 2.66±0.49 | 1.76±0.28 | UAE5 E90 | 16.41±1.56 | 4.43±0.69 | 2.81±0.47 | MAE3 E90 | 25.66±1.96 | 5.35±0.96 | 3.56±0.34 |
| CSLE30 E100 | 8.96±1.09 | 2.45±0.45 | 1.49±0.19 | UAE5 E100 | 16.10±1.52 | 4.09±0.52 | 2.33±0.45 | MAE3 E100 | 24.59±1.84 | 5.13±0.89 | 3.42±0.36 |
| CSLE30 M60 | 8.86±1.02 | 2.37±0.41 | 1.50±0.20 | UAE5 M60 | 14.86±1.24 | 4.14±0.50 | 2.50±0.38 | MAE3 M60 | 22.29±1.71 | 4.64±0.91 | 3.10±0.29 |
| CSLE30 M70 | 9.02±1.12 | 2.56±0.51 | 1.68±0.18 | UAE5 M70 | 15.41±1.39 | 4.27±0.53 | 2.68±0.47 | MAE3 M70 | 24.92±1.53 | 5.19±0.74 | 3.46±0.38 |
| CSLE30 M90 | 8.96±1.28 | 2.47±0.43 | 1.63±0.21 | UAE5 M90 | 15.33±1.36 | 4.22±0.51 | 2.61±0.45 | MAE3 M90 | 24.30±1.49 | 5.06±0.71 | 3.38±0.33 |
| CSLE30 M100 | 8.31±1.19 | 2.30±0.33 | 1.48±0.16 | UAE5 M100 | 14.59±1.25 | 3.98±0.47 | 2.29±0.40 | MAE3 M100 | 21.98±1.21 | 4.58±0.63 | 3.05±0.27 |
| CSLE45 E60 | 10.55±1.31 | 2.76±0.53 | 1.65±0.26 | UAE10 E60 | 14.37±1.21 | 4.05±0.49 | 2.45±0.42 | MAE5 E60 | 22.96±1.34 | 4.79±0.79 | 3.19±0.31 |
| CSLE45 E70 | 10.72±1.36 | 2.93±0.55 | 1.88±0.30 | UAE10 E70 | 14.76±1.29 | 4.27±0.54 | 2.88±0.49 | MAE5 E70 | 26.98±1.96 | 5.62±0.89 | 3.75±0.48 |
| CSLE45 E90 | 10.67±1.39 | 2.88±0.51 | 1.83±0.29 | UAE10 E90 | 14.67±1.31 | 4.22±0.52 | 2.79±0.51 | MAE5 E90 | 26.15±1.99 | 5.45±0.92 | 3.63±0.47 |
| CSLE45 E100 | 10.47±1.41 | 2.66±0.39 | 1.51±0.27 | UAE10 E100 | 14.22±1.18 | 3.89±0.38 | 2.39±0.38 | MAE5 E100 | 22.40±1.64 | 4.66±0.77 | 3.11±0.28 |
| CSLE45 M60 | 9.66±1.33 | 2.69±0.41 | 1.63±0.26 | UAE10 M60 | 14.06±1.14 | 3.75±0.35 | 2.38±0.34 | MAE5 M60 | 21.04±1.55 | 4.38±0.62 | 2.92±0.24 |
| CSLE45 M70 | 10.02±1.37 | 2.77±0.46 | 1.74±0.29 | UAE10 M70 | 14.32±1.23 | 4.06±0.41 | 2.66±0.41 | MAE5 M70 | 22.77±1.64 | 4.75±0.78 | 3.16±0.34 |
| CSLE45 M90 | 9.97±1.33 | 2.74±0.48 | 1.70±0.27 | UAE10 M90 | 14.22±1.17 | 3.92±0.36 | 2.60±0.37 | MAE5 M90 | 22.37±1.67 | 4.66±0.64 | 3.11±0.29 |
| CSLE45 M100 | 9.48±1.29 | 2.59±0.34 | 1.49±0.23 | UAE10 M100 | 13.96±1.13 | 3.65±0.37 | 2.35±0.24 | MAE5 M100 | 19.85±1.29 | 4.14±0.59 | 2.76±0.22 |
| CSLE60 E60 | 8.03±1.25 | 2.18±0.28 | 1.39±0.31 | UAE15 E60 | 13.85±1.11 | 3.77±0.39 | 2.39±0.26 | MAE8 E60 | 20.67±1.33 | 4.31±0.49 | 2.87±0.24 |
| CSLE60 E70 | 8.20±1.41 | 2.31±0.24 | 1.55±0.25 | UAE15 E70 | 14.14±1.27 | 3.98±0.36 | 2.67±0.39 | MAE8 E70 | 23.05±1.46 | 4.80±0.52 | 3.20±0.58 |
| CSLE60 E90 | 8.15±1.23 | 2.27±0.25 | 1.52±0.29 | UAE15 E90 | 14.05±1.24 | 3.92±0.40 | 2.61±0.37 | MAE8 E90 | 22.57±1.28 | 4.70±0.49 | 3.14±0.46 |
| CSLE60 E100 | 7.93±1.26 | 2.10±0.21 | 1.30±0.27 | UAE15 E100 | 13.67±1.13 | 3.62±0.33 | 2.24±0.26 | MAE8 E100 | 19.39±1.14 | 4.04±0.35 | 2.70±0.28 |
| CSLE60 M60 | 7.71±1.35 | 2.08±0.18 | 1.34±0.24 | UAE15 M60 | 13.28±1.06 | 3.59±0.31 | 2.30±0.24 | MAE8 M60 | 19.91±1.11 | 4.15±0.37 | 2.77±0.37 |
| CSLE60 M70 | 7.93±1.16 | 2.18±0.22 | 1.45±0.27 | UAE15 M70 | 13.67±1.24 | 3.77±0.35 | 2.50±0.28 | MAE8 M70 | 21.62±1.37 | 4.50±0.46 | 3.00±0.36 |
| CSLE60 M90 | 7.87±1.41 | 2.16±0.28 | 1.42±0.24 | UAE15 M90 | 13.57±1.19 | 3.73±0.34 | 2.44±0.25 | MAE8 M90 | 21.10±1.42 | 4.39±0.47 | 2.93±0.26 |
| CSLE60 M100 | 7.61±1.34 | 2.00±0.23 | 1.28±0.29 | UAE15 M100 | 13.12±1.02 | 3.45±0.29 | 2.20±0.21 | MAE8 M100 | 19.01±1.14 | 3.96±0.34 | 2.64±0.37 |
| CSLE90 E60 | 6.83±1.01 | 1.74±0.22 | 1.36±0.28 | UAE20 E60 | 12.41±0.92 | 3.16±0.22 | 2.48±0.27 | MAE10 E60 | 20.49±1.03 | 4.27±0.51 | 2.85±0.44 |
| CSLE90 E70 | 6.96±1.11 | 1.86±0.27 | 1.39±0.22 | UAE20 E70 | 12.66±0.98 | 3.39±0.24 | 2.53±0.29 | MAE10 E70 | 20.95±1.07 | 4.37±0.56 | 2.91±0.36 |
| CSLE90 E90 | 6.91±0.98 | 1.83±0.34 | 1.38±0.27 | UAE20 E90 | 12.57±0.96 | 3.32±0.23 | 2.50±0.30 | MAE10 E90 | 20.67±0.99 | 4.31±0.55 | 2.87±0.49 |
| CSLE90 E100 | 6.75±0.94 | 1.67±0.35 | 1.23±0.19 | UAE20 E100 | 12.28±0.89 | 2.98±0.19 | 2.20±0.20 | MAE10 E100 | 18.22±0.88 | 3.80±0.64 | 2.53±0.21 |
| CSLE90 M60 | 6.35±1.08 | 1.59±0.28 | 1.22±0.18 | UAE20 M60 | 11.54±1.06 | 2.92±0.18 | 2.23±0.18 | MAE10 M60 | 18.25±1.19 | 3.84±0.68 | 2.55±0.26 |
| CSLE90 M70 | 6.50±1.07 | 1.75±0.24 | 1.25±0.17 | UAE20 M70 | 11.82±1.04 | 3.18±0.33 | 2.28±0.23 | MAE10 M70 | 18.90±0.96 | 3.94±0.75 | 2.62±0.33 |
| CSLE90 M90 | 6.47±1.02 | 1.71±0.18 | 1.24±0.16 | UAE20 M90 | 11.76±0.88 | 3.10±0.34 | 2.26±0.24 | MAE10 M90 | 18.72±0.87 | 3.90±0.77 | 2.60±0.51 |
| CSLE90 M100 | 6.24±1.12 | 1.55±0.15 | 1.21±0.17 | UAE20 M100 | 11.35±0.93 | 2.81±0.26 | 2.15±0.19 | MAE10 M100 | 17.76±0.83 | 3.70±0.46 | 2.47±0.55 |
